# Released Parasite-Derived Kinases as Novel Targets for Antiparasitic Therapies

**DOI:** 10.3389/fcimb.2022.825458

**Published:** 2022-02-17

**Authors:** Anne Silvestre, Sharvani Shrinivas Shintre, Najma Rachidi

**Affiliations:** ^1^ INRAE, Université de Tours, ISP, Nouzilly, France; ^2^ Institut Pasteur, Université de Paris and INSERM U1201, Unité de Parasitologie Moléculaire et Signalisation, Paris, France

**Keywords:** excreted kinase, eukaryote, protozoa, signal transduction, antimicrobial therapy

## Abstract

The efficient manipulation of their host cell is an essential feature of intracellular parasites. Most molecular mechanisms governing the subversion of host cell by protozoan parasites involve the release of parasite-derived molecules into the host cell cytoplasm and direct interaction with host proteins. Among these released proteins, kinases are particularly important as they govern the subversion of important host pathways, such as signalling or metabolic pathways. These enzymes, which catalyse the transfer of a phosphate group from ATP onto serine, threonine, tyrosine or histidine residues to covalently modify proteins, are involved in numerous essential biological processes such as cell cycle or transport. Although little is known about the role of most of the released parasite-derived kinases in the host cell, they are examples of kinases hijacking host cellular pathways such as signal transduction or apoptosis, which are essential for immune response evasion as well as parasite survival and development. Here we present the current knowledge on released protozoan kinases and their involvement in host-pathogen interactions. We also highlight the knowledge gaps remaining before considering those kinases - involved in host signalling subversion - as antiparasitic drug targets.

## Introduction

The efficient manipulation of their host cell is an essential feature of intracellular parasites, which they achieve by secreting effectors to maintain their replicative niche within the host cell and to hijack important host pathways. Among those effectors, kinases have been shown to regulate a wide range of pathways, such as signalling or metabolic pathways. The potential key role of secreted kinases in the subversion of host cell signalling pathways, make them candidates of choice for the development of new antiparasitic treatments, particularly with the growing concern of drug resistance. Targeting secreted effectors may reduce the risk of drug resistance, as any mutation to bypass the drug effect may prevent their extra-parasite role and thus result in high fitness costs for the parasite. Despite their importance, only few parasite-secreted kinases have been studied and their functions in the host cell characterised. We focused on two phylums:

-The Apicomplexa (Alveolata) with ***Plasmodium* spp.*, Toxoplasma gondii*** and ***Eimeria tenella***, the causative agents of malaria - transmitted by female anopheles mosquitoes -, toxoplasmosis and coccidiosis, respectively;
-The kinetoplastids (Euglenozoa) with ***Leishmania* spp.**, the causative agent of leishmaniasis, transmitted by the bite of a female sand fly and ***Trypanosoma cruzi*,** the causative agent of Chagas disease, spread by Triatominae.

In the present mini review, we describe the known mechanisms of parasite effector secretion and compare the secreted kinome from phylogenetically distant intracellular protozoan parasites. We give examples of the host functions of the few studied secreted kinases and highlight the scientific gaps remaining to fully understand host signalling subversion by parasites.

## Mechanisms Of Parasite Effector Release/Secretion

Parasites release virulence factors either as soluble molecules or inside extracellular vesicles (EVs), leading to the modification of the biological and immune functions of their host cell to ensure their survival (Silverman et al., 2010b; Regev-Rudzki et al., 2013). Apicomplexa and kinetoplastids parasites display different mode of host-parasite interactions due to the specificity of their host cell and their mode of cell entry, which might be partly reflected in the mechanism of parasite protein secretion.

### Common to Most Apicomplexa and Kinetoplastids

Exocytosis, driven by the active transport of secretory vesicles is the eukaryotic conventional secretion system for proteins containing a hydrophobic domain in N-terminal position [Signal Peptide, SP, see (Beer and Wehman, 2017) for an illustrated review]. These protein-containing vesicles traffic from the Golgi apparatus to the plasma membrane, fuse with the plasma membrane to release the secreted proteins in the extracellular environment (Colombo et al., 2014). This secretion system does not account for all the molecules exported by parasites. In *Leishmania*, 98% of the secreted proteins lack SP, suggesting the presence of other secretion pathways (Silverman et al., 2008). The second mechanism is unconventional protein secretion (UPS) and refers to proteins either exposed on the cell surface or in the extracellular medium (Balmer and Faso, 2021). The third mechanism is through extracellular vesicles (EVs), lipid-bound vesicles which either bud from the plasma membrane (microvesicles) or are derived from multivesicular bodies that fuse with the plasma membrane (exosomes) (Dlugonska and Gatkowska, 2016; Mathieu et al., 2019; Babatunde et al., 2020) and for more details in mechanisms of secretion see (Teng and Fussenegger, 2021).

### Specific to Apicomplexa

Apicomplexa have developed specific strategies to release proteins into the host cell, which might be the consequence of cell entry by invasion, contrary to phagocytosis or endocytosis for kinetoplastids. Cell invasion requires the fast discharge of microneme and rhoptry proteins (perforins, lipases, proteases, adhesins and kinases) implicated in gliding motility, parasite attachment, formation of the moving junction and the hijacking of host cell pathways, which might not be compatible with the slower release of proteins by the secretory or exosomal pathways (Tomavo et al., 2013; Bisio and Soldati-Favre, 2019). Proteins are targeted to those compartments by conventional SP and specific motifs. Microneme secretion is triggered by signalling events, involving intracellular cyclic nucleotides, calcium level and phosphatidic acid (Dubois and Soldati-Favre, 2019) and is followed by rhoptry secretion (Aquilini et al., 2021). In addition to micronemes and rhoptries, *Plasmodium* species create, in the host cell cytoplasm, a network of membranous structures of parasite origin, called Maurer’s clefts. These structures are attached to the host cytoskeleton and act as extracellular secretory and trafficking organelles for the parasite (Lanzer et al., 2006) but little is known about their biogenesis and functions. Finally, some proteins contain PEXEL motifs in *Plasmodium* (Marti et al., 2004) and TEXEL motifs in *Toxoplasma* (Coffey et al., 2015), which are required for their release *via* exporters located on the parasitophorous vacuole membrane (PVM). The PVM derives from the host cell membrane and is modified by incorporation of parasite proteins, to avoid phagolysosome fusion (de Koning-Ward et al., 2009; Marino et al., 2018). This pathway corresponds to the default constitutive secretion pathway (Venugopal et al., 2020).

## Exoproteomes of Apicomplexa and Trypanosomatids

There is a growing body of data on the exo-proteome, whatever the mechanism of secretion used by parasites. It contains a range of protein classes including proteases, kinases, membrane proteins, heat shock proteins or nucleic acids, which induce specific modifications in the host cell (Montaner et al., 2014). Only little is known about the mechanisms involved in cargo selection of these EVs. *Leishmania* HSP100 has a strong impact on protein cargo composition: its deletion affects the immune status of the host cell and parasite survival (Silverman et al., 2011). The EV composition is sensitive to environmental cues (Hassani et al., 2011) and might contribute to the spread of drug resistance (Douanne et al., 2020). EVs have an essential role during infection (Torrecilhas et al., 2020); co-egestion of *Leishmania* and its EVs by the sand fly induces the inflammatory recruitment of neutrophils and macrophages (Atayde et al., 2015). EVs are involved in immune evasion; *T. cruzi* exosomes aggravate the infection due to severe inflammatory response and increase the parasite burden (Trocoli Torrecilhas et al., 2009). Several vesicular virulence factors from *T. cruzi* have been involved in host invasion, intracellular parasite proliferation or immune evasion (Costa et al., 2016). For Apicomplexa, *T. gondii* exosomes has been shown to activate a pro-inflammatory immune response (Li et al., 2018), and small non-coding RNAs and genomic DNA contained in EVs released from RBC infected by *P. falciparum* are detected by the STING pathway, favouring parasite survival (Sisquella et al., 2017). However, accessing parasite-derived EVs is challenging, as apicomplexans parasites cannot be cultured without their host cell, thus most of the data available on their exo-proteome is in fact from host-derived EVs.

## Secreted/Excreted Parasite Kinases

Phosphorylation, an essential reversible post-translational modification, affects every cellular process (Ardito et al., 2017). It acts as a molecular switch for many biological processes, including signal transduction networks in response to extracellular stimuli. Phosphorylation is catalysed by kinases, which transfers phosphate from ATP onto proteins, sugars or lipids. Upon phosphorylation, the chemical properties, conformation, localisation and/or activity of the molecule change, inducing rapid downstream effects in the cell (Hunter and Sibley, 2012). To survive, intracellular pathogens need to exploit the host pathways either to fulfil their needs for proliferation or to inhibit the host defence responses. Targeting the phospho-proteome of the host is the fastest way to subvert a large repertoire of biological and immune processes (Regev-Rudzki et al., 2013; Carrera-Bravo et al., 2021).

### Kinases

Most studies on kinases refers to protein kinases. Protozoan parasite kinomes contain orthologues for 6 of the 8 groups of conventional eukaryotic PKs (ePK): AGC, CAMK, CK1, CMGC, STE and TKL) and some “others” that share ePK folding but cannot be assigned to any major ePK group from humans (Peixoto et al., 2010; Talevich et al., 2011). One additional group Kinetoplastids, NEK family, is involved in cell cycle and cytoskeletal functions. Apicomplexa contains also specific ePK (FIKK, ROPK and WNG), differentially conserved and/or duplicated within Apicomplexa parasite phylum (Ward et al., 2004; Beraki et al., 2019). While only one FIKK gene was identified in coccidia (*Toxoplasma, Eimeria*) and in most *Plasmodium* species, this group is expanded in *P. falciparum* with 20 kinases and in several *Plasmodium* sp. infecting apes (Adderley et al., 2021). Most of the rhoptry proteins are kinases (ROPK), either active, inactive (lacking a complete catalytic triad) or non-canonical (active with differences in conserved residues) (Bradley et al., 2005). Finally, very recently, a new group of four kinases specific to coccidia, and missing the typical glycine loop was identified (WNG1-3 and BPK1) (Beraki et al., 2019). BPK1 is associated with bradyzoite cyst wall, with a crucial role in *in vivo* cyst infectivity (Boothroyd, 2013; Buchholz et al., 2013). In *Eimeria*, two WNGs are predicted, but their functions remain unknown. To date, only TgWNG1 has been functionally characterized: it is involved in the phosphorylation of GRA, a family of effectors stored in dense granule vesicles and secreted to develop the intra-vacuolar network, implicated in survival of parasite. Although as important as protein kinases, there are no comprehensive studies available on carbohydrate, lipid, nucleoside or other kinases, but only individual publications (Pereira et al., 2011).

### Host Functions of Secreted Kinases

**Figure 1:**
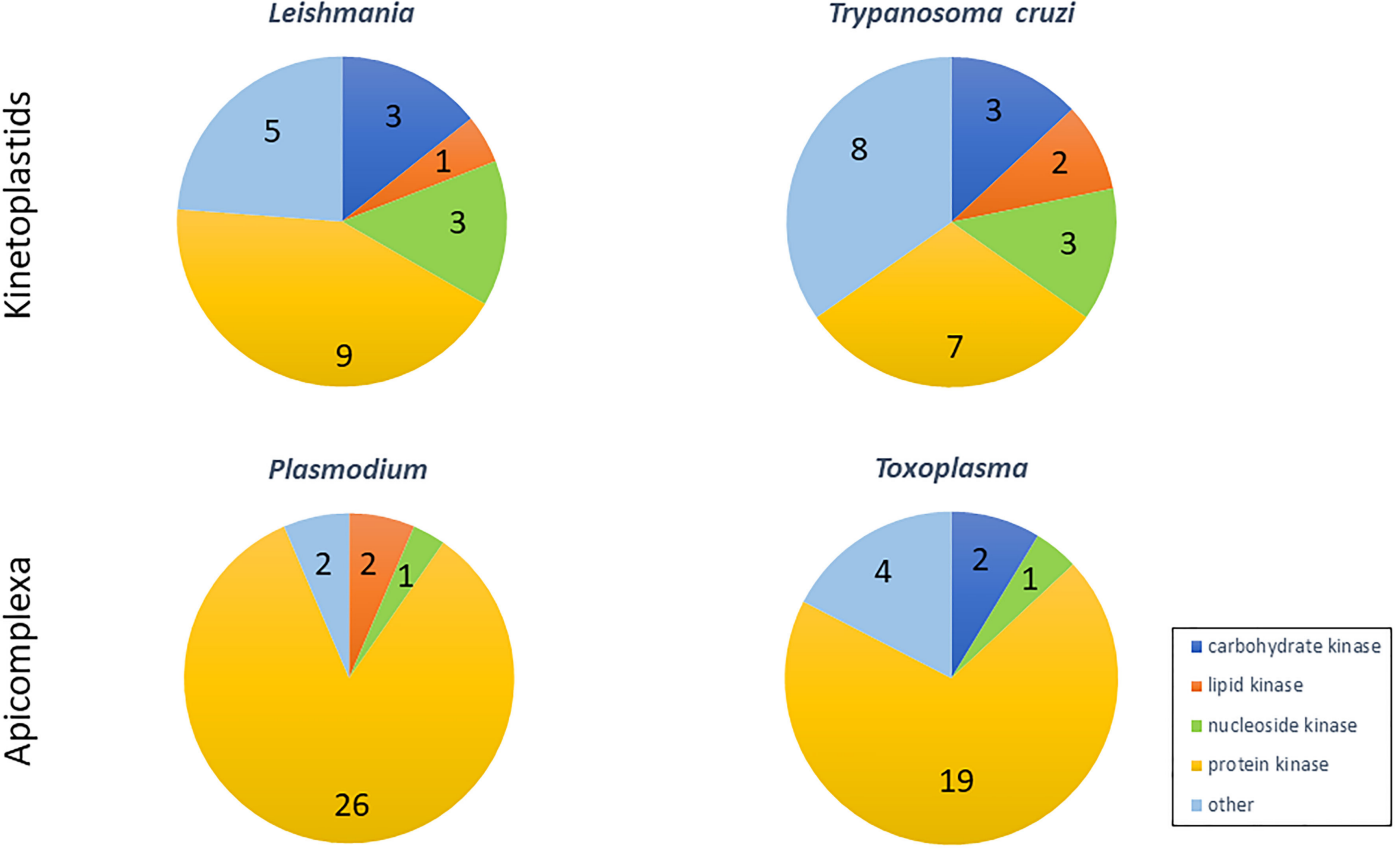
Proportion and number of kinases classes according to their substrates, identified in secretome of Kinetoplastids (*Leishmania* and *Trypanozoma*) and Apicomplexa (*Toxoplasma* and *Plasmodium*).

Proteomic characterisation of parasite exo-proteomes revealed the presence of only few kinases, mostly involved in pathways such as glycolysis, cellular energy homeostasis or cell signalling (**Table 1**). While in Apicomplexa most secreted kinases target proteins, in *Leishmania* and *T. cruzi*, more than half of the kinases target nucleosides, carbohydrates or lipids (**Figure 1**). Eight kinases are released by both kinetoplastids and Apicomplexa (**Table 1**, bold), suggesting that host subversion mediated by those kinases might be conserved between parasites. Five kinases might be kinetoplastid-specific (**Table 1**, italic) and only one might be Apicomplexa-specific (CDPKs, **Table 1**, underlined). This low number of specific kinases may be due to the scarce proteomic data available for these parasites.

**Table 1:** Kinases secreted by protozoan parasites.

Organism	Kinase Name	Kinase Class	References	Stages
**Leishmania**	6-phospho-1-fructokinase, putative	carbohydrate	Silverman et al., 2008; Silverman et al., 2010a; Douanne et al., 2020	Stationary-phase promastigotes, logarithmic promastigotes
	*adenosine kinase, putative*	nucleoside	Silverman et al., 2008	Stationary-phase promastigotes
	adenylate kinase, putative	nucleoside	Silverman et al., 2008; Silverman et al., 2010a; Hassani et al., 2011; Douanne et al., 2020	Stationary-phase promastigotes, logarithmic promastigotes and axenic amastigotes
	**casein kinase I, putative CK1.2**	protein	Silverman et al., 2008; Silverman et al., 2010a	Stationary-phase promastigotes
	**casein kinase II, alpha chain, Putative LmjF.02.0360**	protein	Douanne et al., 2020	Logarithmic promastigotes
	*mevalonate kinase*	lipid	Bamra et al., 2021	Promastigotes and amastigotes
	cdc2-related kinase 9	protein	Silverman et al., 2008	Stationary-phase promastigotes
	glycogen synthase kinase3-putative	protein	Douanne et al., 2020	Logarithmic promastigotes
	**glycosomal phosphoenolpyruvate carboxykinase, putative**	other	Silverman et al., 2008; Hassani et al., 2011; Douanne et al., 2020	Stationary-phase promastigotes and logarithmic promastigotes
	**hexokinase, putative**	carbohydrate	Silverman et al., 2008; Hassani et al., 2011	Stationary-phase promastigotes
	*mitogen activated protein kinase, putative,map kinase, putative*	protein	Silverman et al., 2008	Stationary-phase promastigotes
	*mitogen-activated protein kinase 3, putative,map kinase 3, putative*	protein	Silverman et al., 2008	Stationary-phase promastigotes
	**nucleoside diphosphate kinase b**	nucleoside	Silverman et al., 2008; Silverman et al., 2010a; Hassani et al., 2011; Douanne et al., 2020	Stationary-phase promastigotes, logarithmic promastigotes and axenic amastigotes.
	**phosphoglycerate kinase**	other	Douanne et al., 2020; Mandacaru et al., 2019; Ribeiro et al., 2018; Brossas et al., 2017; Queiroz et al., 2016; Bayer-Santos et al., 2013; Hassani et al., 2011	Logarithmic promastigotes, stationary-phase promastigotes and Epimastigotes, Vero cells infected with trypomastigotes, Trypomastigotes, Tissue culture-derived trypomastigotes and trypomastigotes.
	protein kinase, putative LmjF34.0030	protein	Silverman et al., 2008	Stationary-phase promastigotes
	pyruvate dehydrogenase lipoamide kinase, putative	other	Silverman et al., 2008	Stationary-phase promastigotes
	**pyruvate kinase, putative**	other	Silverman et al., 2008; Silverman et al., 2010a; Douanne et al., 2020; Ribeiro et al., 2018	Stationary-phase promastigotes and logarithmic promastigotes, Tissue culture-derived trypomastigotes.
	*pyruvate phosphate dikinase, putative*	other	Silverman et al., 2008; Silverman et al., 2010a; Douanne et al., 2020	Stationary-phase promastigotes and logarithmic promastigotes
	*serine/threonine-protein kinase, putative LINF_290033500/LmjF29.2570 identity to human Nek1*	protein	Douanne et al., 2020	Logarithmic promastigotes
	serine/threonine-protein kinase, putative LmjF25.2340 identity to human AKT1	protein	Silverman et al., 2010a	Stationary-phase promastigotes
	tagatose-6-phosphate kinase-like protein	carbohydrate	Silverman et al., 2008	Stationary-phase promastigotes
**Trypanosoma cruzi**	*adenosine kinase, putative*	nucleoside	Brossas et al., 2017	Trypomastigotes
	*mevalonate kinase*	lipid	Ferreira et al., 2016	Metacyclic trypomastigote and extracellular amastigote cultures
	adenylate kinase, putative	nucleoside	Queiroz et al., 2016; Ribeiro et al., 2018	Trypomastigotes, Tissue culture-derived trypomastigotes
	arginine kinase	other	Bayer-Santos et al., 2013; Queiroz et al., 2016; Brossas et al., 2017; Ribeiro et al., 2018; Mandacaru et al., 2019	Epimastigotes, Vero cells infected with trypomastigotes, Trypomastigotes, Tissue culture-derived trypomastigotes and trypomastigotes.
	fucose kinase	carbohydrate	Ribeiro et al., 2018	Tissue culture-derived trypomastigotes
	galactokinase	carbohydrate	Ribeiro et al., 2018	Tissue culture-derived trypomastigotes
	**glycosomal phosphoenolpyruvate carboxykinase, putative**	other	Queiroz et al., 2016; Ribeiro et al., 2018; Mandacaru et al., 2019	Trypomastigotes, Tissue culture-derived trypomastigotes and trypomastigotes.
	**hexokinase**	carbohydrate	Bayer-Santos et al., 2013, Mandacaru et al., 2019	Epimastigotes and trypomastigotes.
	mitogen-activated protein kinase, putative	protein	Bayer-Santos et al., 2013; Brossas et al., 2017; Ribeiro et al., 2018	Epimastigotes, Vero cells infected with trypomastigotes, Tissue culture-derived trypomastigotes
	*NIMA-related kinase, putative*	protein	Queiroz et al., 2016	Trypomastigotes
	**nucleoside diphosphate kinase B**	nucleoside	Bayer-Santos et al., 2013; Brossas et al., 2017; Queiroz et al., 2016; Ribeiro et al., 2018; Mandacaru et al., 2019	Epimastigotes, Vero cells infected with trypomastigotes, Trypomastigotes, Tissue culture-derived trypomastigotes and trypomastigotes.
	phosphatidylinositol-3-Kinase	lipid	Bayer-Santos et al., 2013	Epimastigotes
	Protein kinase Tc00.1047053506211.210 MAPKKK identity to human PAK1/PAK3	protein	Ribeiro et al., 2018	Tissue culture-derived trypomastigotes
	Protein kinase, putative TcCLB.508641.170 identity to human PKC theta	protein	Brossas et al., 2017	Vero cells infected with trypomastigotes
	protein kinase-A catalytic subunit	protein	Queiroz et al., 2016	Trypomastigotes
	pyridoxal kinase, putative	other	Queiroz et al., 2016; Ribeiro et al., 2018	Trypomastigotes, Tissue culture-derived trypomastigotes
	**pyruvate kinase 2, putative**	other	Queiroz et al., 2016	Trypomastigotes
	*pyruvate phosphate dikinase 2*	other	Queiroz et al., 2016	Trypomastigotes
	*pyruvate phosphate dikinase 1*	other	Bayer-Santos et al., 2013; Ribeiro et al., 2018	Epimastigotes, Tissue culture-derived trypomastigotes
	serine/threonine protein kinase *TcCLB.508153.400/TCSYLVIO_004423 identity to human Nek1*	protein	Bayer-Santos et al., 2013; Ribeiro et al., 2018	Epimastigotes, Tissue culture-derived trypomastigotes
	serine/threonine-protein kinase 10, putative TcCLB.506401.110 identity to human STK10	protein	Queiroz et al., 2016	Trypomastigotes
**Plasmodium**	Camk2d calcium/calmodulin_dependent protein kinase II_ delta isoform 1	protein	Martin-Jaular et al., 2011	Trophozoite *P. yoelii* infected reticulocyte
	Camk2d Isoform 1 of Calcium/calmodulin_dependent protein kinase type II delta chain	protein	Martin-Jaular et al., 2011	Trophozoite *P. yoelii* infected reticulocyte
	Camk2d Isoform 2 of Calcium/calmodulin_dependent protein kinase type II delta chain	protein	Martin-Jaular et al., 2011	Trophozoite *P. yoelii* infected reticulocyte
	Camk2d Isoform 3 of Calcium/calmodulin_dependent protein kinase type II delta chain	protein	Martin-Jaular et al., 2011	Trophozoite *P. yoelii* infected reticulocyte
	Camk2d Isoform 4 of Calcium/calmodulin_dependent protein kinase type II delta chain	protein	Martin-Jaular et al., 2011	Trophozoite *P. yoelii* infected reticulocyte
	**casein kinase 2, alpha subunit**	protein	Abdi et al., 2017	Trophozoite *P. falciparum* infected erythrocyte
	diacyl glycerol kinase	lipid	Gualdron-Lopez et al., 2018	Trophozoite *P. vivax* infected erythrocyte
	FIKK10.1	protein	Hiller et al., 2004	Trophozoite *P. falciparum* infected erythrocyte
	FIKK13	protein	Hiller et al., 2004	Trophozoite *P. falciparum* infected erythrocyte
	FIKK14	protein	Hiller et al., 2004	Trophozoite *P. falciparum* infected erythrocyte
	FIKK1	protein	Hiller et al., 2004	Trophozoite *P. falciparum* infected erythrocyte
	FIKK4.1	protein	Hiller et al., 2004	Trophozoite *P. falciparum* infected erythrocyte
	FIKK4.2	protein	Hiller et al., 2004	Trophozoite *P. falciparum* infected erythrocyte
	FIKK7.1	protein	Hiller et al., 2004	Trophozoite *P. falciparum* infected erythrocyte
	FIKK9.1	protein	Hiller et al., 2004	Trophozoite *P. falciparum* infected erythrocyte
	FIKK9.3	protein	Hiller et al., 2004	Trophozoite *P. falciparum* infected erythrocyte
	FIKK9.6	protein	Hiller et al., 2004	Trophozoite *P. falciparum* infected erythrocyte
	FIKK10.2	protein	Hiller et al., 2004	Trophozoite *P. falciparum* infected erythrocyte
	FIKK11	protein	Hiller et al., 2004	Trophozoite *P. falciparum* infected erythrocyte
	FIKK12	protein	Hiller et al., 2004	Trophozoite *P. falciparum* infected erythrocyte
	pacsin2 Protein kinase C and casein kinase substrate in neurons protein 2	protein	Martin-Jaular et al., 2011	Trophozoite *P. yoelii* infected reticulocyte
	phosphatidylinositol 4-kinase, putative	lipid	Abdi et al., 2017	Trophozoite *P. falciparum* infected erythrocyte
	**phosphoglycerate kinase**	other	Mantel et al., 2013; Abdi et al., 2017	Trophozoite *P. falciparum* infected erythrocyte
	pseudo protein kinase 1, putative PF3D7_0321400	protein	Abdi et al., 2017	Trophozoite *P. falciparum* infected erythrocyte
	**pyruvate kinase**	other	Vincensini et al., 2005; Mantel et al., 2013	Trophozoite *P. falciparum* infected erythrocyte
	serine/threonine protein kinase, putative PF3D7_1441300	protein	Abdi et al., 2017	Trophozoite *P. falciparum* infected erythrocyte
	calcium-dependent protein kinase CDPK1	protein	Lal et al., 2009	Purified microneme organelle
	calcium-dependent protein kinase CDPK4	protein	Lal et al., 2009	Purified microneme organelle
	adenylate kinase	nucleoside	Vincensini et al., 2005	Trophozoite *P. falciparum* infected erythrocyte
**Toxoplasma**	calcium-dependent protein kinase CDPK1	protein	Wowk et al., 2017; Ramirez-Flores et al., 2019	Tachyzoite *T. gondii* infected human foreskin fibroblast, Acellular tachyzoites
	calcium-dependent protein kinase CDPK2A	protein	Wowk et al., 2017	Tachyzoite *T. gondii* infected human foreskin fibroblast
	calcium-dependent protein kinase CDPK3	protein	Wowk et al., 2017; Ramirez-Flores et al., 2019	Tachyzoite *T. gondii* infected human foreskin fibroblast, Acellular tachyzoites
	**casein kinase I**	protein	Wowk et al., 2017	Tachyzoite *T. gondii* infected human foreskin fibroblast
	CMGC kinase, CK2 family	protein	Wowk et al., 2017; Ramirez-Flores et al., 2019	Tachyzoite *T. gondii* infected human foreskin fibroblast, Acellular tachyzoites
	**hexokinase**	carbohydrate	Wowk et al., 2017; Ramirez-Flores et al., 2019	Tachyzoite *T. gondii* infected human foreskin fibroblast, Acellular tachyzoites
	**nucleoside diphosphate kinase**	nucleoside	Lee et al., 2014	Acellular tachyzoites
	**phosphoenolpyruvate-carboxykinase I**	other	Wowk et al., 2017	Tachyzoite *T. gondii* infected human foreskin fibroblast
	phosphofructokinase PFKII	carbohydrate	Wowk et al., 2017	Tachyzoite *T. gondii* infected human foreskin fibroblast
	**phosphoglycerate kinase**	other	Wowk et al., 2017	Tachyzoite *T. gondii* infected human foreskin fibroblast
	**pyruvate kinase**	other	Wowk et al., 2017	Tachyzoite *T. gondii* infected human foreskin fibroblast
	rhoptry kinase family protein ROP39	protein	Wowk et al., 2017	Tachyzoite *T. gondii* infected human foreskin fibroblast
	selenide, water dikinase	other	Wowk et al., 2017	Tachyzoite *T. gondii* infected human foreskin fibroblast
	ROP2 - PK-like	protein	Bradley et al., 2005	Purified rhoptry organelle
	ROP4 - PK-like	protein	Bradley et al., 2005	Purified rhoptry organelle
	ROP5 - PK-like	protein	Bradley et al., 2005	Purified rhoptry organelle
	ROP8 - PK-like	protein	Bradley et al., 2005	Purified rhoptry organelle
	ROP11 - PK-like	protein	Bradley et al., 2005	Purified rhoptry organelle
	ROP16 - PK-like	protein	Bradley et al., 2005	Purified rhoptry organelle
	ROP17 - PK-like	protein	Bradley et al., 2005	Purified rhoptry organelle
	ROP18 - PK-like	protein	Bradley et al., 2005	Purified rhoptry organelle
	ROP38 - PK-like	protein	Bradley et al., 2005	Purified rhoptry organelle
	WNG1 (With-No-Gly-Loop)	protein	Beraki et al., 2019	Bradyzoite
	WNG2	protein	Beraki et al., 2019	Bradyzoite
	WNG3	protein	Beraki et al., 2019	Bradyzoite
	BPK1 bradyzoite pseudokinase 1	protein	Buchholz et al., 2013	Bradyzoite
Eimeria^a^	**pyruvate kinase**	other	Labbé et al., 2006	Schizont from *in vitro* infected cells
	**hexokinase**	carbohydrate	Sun et al., 2016	Sporozoite from *in vitro* infected cells
	calcium-dependent protein kinase CDPK1	protein	Dunn et al., 1996	Sporozoite from *in vitro* infected cells
	calcium-dependent protein kinase CDPK2	protein	Dunn et al., 1996	Sporozoite from *in vitro* infected cells
	calcium-dependent protein kinase CDPK3	protein	Han et al., 2013	Sporozoite and schizont from *in vitro* infected cells
	calcium-dependent protein kinase CDPK4	protein	Wang et al., 2016a	Sporozoite and merozoite from *in vitro* infected cells
	ETH_00000075	protein	Oakes et al., 2013	Purified rhoptry organelle
	ETH_00005190 - EtROP1	protein	Oakes et al., 2013	Purified rhoptry organelle
	ETH_00005400	protein	Oakes et al., 2013	Purified rhoptry organelle
	ETH_00005840	protein	Oakes et al., 2013	Purified rhoptry organelle
	ETH_00005905	protein	Oakes et al., 2013	Purified rhoptry organelle
	ETH_00020620	protein	Oakes et al., 2013	Purified rhoptry organelle
	ETH_00026495	protein	Oakes et al., 2013	Purified rhoptry organelle
	ETH_00027695	protein	Oakes et al., 2013	Purified rhoptry organelle
	ETH_00027700	protein	Oakes et al., 2013	Purified rhoptry organelle
	ETH_00027855	protein	Oakes et al., 2013	Purified rhoptry organelle
	ETH_00028765	protein	Oakes et al., 2013	Purified rhoptry organelle
	WNG1, predicted	protein	Beraki et al., 2019	ND^b^
	WNG4, predicted	protein	Beraki et al., 2019	ND^b^

a Secreted kinases listed for Eimeria are underestimated, due to a lack of datasets. Eimeria pyruvate kinase (Labbé et al., 2006), hexokinase (Sun et al., 2016) and CDPK (Dunn et al., 1996; Han et al., 2013; Wang et al., 2016a) are secreted by an unknown mechanism.

b Not determined.

Kinase class refers to the kinase substrate. Kinases common to kinetoplastids and Apicomplexa are indicated in bold, kinases specific to kinetoplastids or to Apicomplexa are indicated in italic or underlined, respectively. Based on experimental procedures of cited references for Apicomplexa, kinases from organelles are secreted in the host cell cytoplasm, not in the extracellular medium. To the author knowledge, only *P. falciparum* TKL2 and PfCK1 were detected in extracellular medium and at the erythrocyte membrane (Abdi et al., 2013) and maybe associated to immunomodulatory functions. Most of the secreted proteins of Kinetoplastids are released inside the host cell. Ndk and AK seem to be secreted in the extracellular environment of the host cell due to their role. Limitations concerning secretome preparation and characterization have been reviewed (Severino et al., 2013).

### Glycolytic Kinases

Glycolytic kinases are located in the glycosomes of kinetoplastids and in the cytosol and the apicoplast of Apicomplexa (Saito et al., 2002; Fleige et al., 2007). They regulate glycolysis but have additional biological functions, as moonlighting proteins. For instance, *Leishmania* hexokinase, a glycolytic enzyme, also acts as a haemoglobin (Hb) receptor, allowing Hb internalisation (Krishnamurthy et al., 2005). *L. donovani* aldolase, another glycolytic enzyme, interacts with and activates the host SHP-1 (protein tyrosine phosphatase). SHP-1 inhibits M1 macrophage polarization, creating a more favourable environment for *Leishmania* (Nandan et al., 2007; Garg et al., 2020). Although important in number, nothing is known about the host functions of glycolytic kinases, but their release by most the parasites suggest important roles in the host cell.

### Nucleoside Diphosphate Kinase (Ndk)

Ndk catalyses the transfer of phosphate from nucleoside triphosphate (NTP) to nucleoside diphosphate (NDP) to maintain ATP cellular homeostasis (Kolli et al., 2008). This kinase also plays roles in the regulation of gene transcription, DNA repair, differentiation and apoptosis (Yu et al., 2017). Ndk seems important for drug resistance in *T. cruzi* and in *Leishmania*, its overexpression leads to a decrease sensitivity to antimony (Sb^III^) (Moreira and Murta, 2016); for DNA damage responses in *T. cruzi* (Miranda et al., 2008); and for parasite replication in *T. gondii* (Lykins et al., 2018). In these parasites, Ndk is released in EVs (Silverman et al., 2008; Silverman et al., 2010a; Bayer-Santos et al., 2013; Lee et al., 2014; Brossas et al., 2017). In *Leishmania*, the release of ndk prevents extracellular ATP (eATP)-mediated cytolysis of infected macrophages (Kolli et al., 2008). eATP, a signal nucleotide, binds to and activates the P2X7 receptor, which is responsible for the pore formation in the membrane of macrophages, resulting in cell death (Kolli et al., 2008; Kulkarni et al., 2019). By transferring phosphate from ATP to NDP, Ndk decreases eATP, thus prevents ATP-induced changes in mitochondrial permeability of macrophages. Furthermore, Ndk participates in the host purine salvage by protozoan parasites by utilizing eATP to produce other NTPs such as GTP (Kolli et al., 2008) These functions might be conserved in *T. cruzi* and *T. gondii*, which also release Ndk.

### Casein Kinase 1

Casein kinase 1 (CK1) is a serine/threonine protein kinase that regulates a wide range of biological processes (Xu et al., 2019; Rachidi et al., 2021). In *Leishmania*, three paralogs are released: L-CK1.4 through the classical secretory pathway, L-CK1.5 and L-CK1.2 *via* exosomes (**Table 1**). Nothing is known about the role of these paralogs in the host cell, except for L-CK1.2. This kinase phosphorylates human IFNAR1 receptor, physiological target of human CK1α, to promote its ubiquitination and subsequent degradation, leading to the attenuation of the cellular response to interferon α/β (Liu et al., 2009). Recently additional host proteins phosphorylated by L-CK1.2 were identified (Smirlis et al., 2022). Several pathways, such as apoptosis, actin skeleton organisation or RNA processing were shown to be potentially regulated by L-CK1.2, which corresponds to pathways altered during *Leishmania* infection (Smirlis et al., 2020). These findings suggest that L-CK1.2 might replace human CK1 and phosphorylate host proteins to modify the immune status of the host cell. Among the three CK1 isoforms encoded by *T. gondii*, only CK1α is secreted in EVs (Donald et al., 2005; Wowk et al., 2017; Rachidi et al., 2021). In contrast to its kinetoplastid orthologs, it is still unclear whether TgCK1α is essential for *T. gondii* survival or what are its functions in the host cell. However, TgCK1α is not a candidate drug target, as its deletion increases *T. gondii* virulence (Wang et al., 2016b). Finally, *P. falciparum* expresses only one CK1, which is secreted by potentially hijacking the trafficking system of the host cell (Dorin-Semblat et al., 2015). Ten PfCK1-interacting host proteins were identified and are involved in various pathways, such as post-translational modifications, translation and protein trafficking/export (Batty et al., 2020).

### Adenylate Kinase (AK)

AK catalyses the transfer of a phosphate group from ATP to AMP to generate two ADPs. It regulates homeostasis of adenine nucleotides and plays an important role in the regulation of the energy metabolism. AK has been detected in the exo-proteome of *Leishmania* and *T. cruzi* (**Table 1**). Recent data from *L. donovani*, suggests that AK2a prevents ATP-mediated cytolysis of macrophages, similarly to Ndk (Kulkarni et al., 2019).

### Mevalonate Kinase

*L. donovani* Mevalonate kinase (MVK) is a glycosomal enzyme, secreted by the parasite *via* a non-classical secretion pathway (Bamra et al., 2021). MVK catalyses the phosphorylation of mevalonic acid into mevalonate-5-phosphate, which is part of the cholesterol biosynthesis pathway. Macrophage infection with *L. donovani* over-expressing MVK leads to an increase in parasite internalisation. During extracellular amastigotes invasion, *T. cruzi* MVK induces the phosphorylation of host Src/FAK, involved in cytoskeleton remodelling of the host (Ferreira et al., 2016), and the phosphorylation of the host P38 and ERK leading to cytoskeleton and microfilament remodelling, which favour parasite internalisation. Moreover, LdMVK is an immuno-suppressor, which favours anti-inflammatory cytokines through ERK1/2, increasing parasite survival (Bamra et al., 2021).

### FIKKs

The 18 FIKKs secreted by *P. falciparum*, display an important non-redundant role in cytoskeletal connections, nutrients permeability and ubiquitination of RBC proteins, as shown by the phosphoproteomic profile of their systematic invalidated mutants (Davies et al., 2020). For instance, FIKK4.1 and FIKK4.2 are involved in cytoadhesion of the RBC to the vascular endothelium, by regulating the number/size of knobs formed on the RBC membrane (Kats et al., 2014). FIKK4.1, FIKK7.1 and FIKK12 phosphorylate host cell cytoskeleton proteins, thus modifying RBC rigidity (Nunes et al., 2010; Brandt and Bailey, 2013). Finally, FIKK9.1, FIKK10.1 and FIKK10.2, exported *via* Maurer’s clefts, are essential for parasite survival (Siddiqui et al., 2020).

### ROPKs

ROPK, secreted from the rhoptries, are involved in host-pathogen interaction. Although not all ROPK are functionally characterized, a systematic and targeted *T. gondii* ROPK knockout screen (Fox et al., 2016) highlighted the role of 20 ROPKs in the establishment of a chronic infection. After their secretion in the host cell cytoplasm, TgROP5, TgROP17 and TgROP18 form a complex on the cytosolic side of the PVM (Etheridge et al., 2014). TgROP5 binds immune-related GTPases (IRG) to decrease their polymerisation rate (Behnke et al., 2012). IRG are then phosphorylated by TgROP18, to prevent their recruitment to the PVM and preserve it (Fleckenstein et al., 2012). Additionally, TgROP17 is also involved in GRA translocation through the PVM, in association with the MYR complex (Panas et al., 2019). TgROP16 (Saeij et al., 2006), TgROP17 (Drewry et al., 2019), TgROP18 (Fentress and Sibley, 2011) and TgROP38 (Peixoto et al., 2010) are known to interfere with and regulate host pathways such as immune response and apoptosis. TgROP16 is localized to the host cell nucleus after invasion (Ong et al., 2010), phosphorylates signal transducer and activator of transcription STAT6 and STAT3 (Yamamoto et al., 2009; Butcher et al., 2011) to bypass the protective immune-response of the host cell. In *E. tenella*, among 28 ROPKs differentially expressed during the life-cycle (Ribeiro E Silva et al., 2021), only EtROP1 has been functionally characterised (Diallo et al., 2019). It interacts with host p53, to inhibit host cell apoptosis and induce G0/G1 cell cycle arrest. Interestingly, EtROP1 kinase activity is only required for cell cycle arrest, supporting the hypothesis of an additional kinase that would be responsible for p53 phosphorylation. ROPK inhibitors may offer new therapeutic treatments to control coccidiosis (Simpson et al., 2016).

## Concluding Remarks

Released divergent kinases that alter host signalling pathways are interesting as they co-evolve with their host targets to insure their proper function within the host and are thus less prone to mutations that would lead to drug resistance. Some compounds that target those secreted kinases have already been identified. *P. falciparum* Pyruvate Kinase is efficiently targeted by antimalarial drugs, such as LZ1 (Fang et al., 2019) or suramin, which also targets trypanosomatids Pyruvate Kinase (Zhong et al., 2020). L-CK1.2 has been validated as a drug target and several compounds with anti-leishmanial activity have been identified, for review see (Rachidi et al., 2021). The similarity between PfCK1 and L-CK1.2 suggests that it might also be a good antimalarial drug target. Given the expanse of their effects on the host cell, understanding the roles that kinases secreted by parasites play in the subversion of host cell signalling will help uncover crucial drug targets.

## Author Contributions

AS and NR wrote the first draft of the manuscript. SS wrote sections of the manuscript. All authors contributed to manuscript revision, read, and approved the submitted version.

## Funding

This work was funded by the French Government (Agence Nationale de la Recherche) Investissement d’Avenir programme, Laboratoire d’Excellence (LabEx) “French Parasitology Alliance For Health Care” (ANR-11-LABX-0024-PARAFRAP) and by the French government, ANR TEXLEISH (ANR-21-CE18-0026).

## Conflict of Interest

The authors declare that the research was conducted in the absence of any commercial or financial relationships that could be construed as a potential conflict of interest.

## Publisher’s Note

All claims expressed in this article are solely those of the authors and do not necessarily represent those of their affiliated organizations, or those of the publisher, the editors and the reviewers. Any product that may be evaluated in this article, or claim that may be made by its manufacturer, is not guaranteed or endorsed by the publisher.
